# Crystal structure of bis­(di­methyl­ammonium) hexa­aqua­cobalt(II) bis­(sulfate) dihydrate

**DOI:** 10.1107/S2056989015003400

**Published:** 2015-03-04

**Authors:** Peter Held

**Affiliations:** a Institut für Kristallographie, Universität zu Köln, Greinstr. 6, D-50939 Köln, Germany

**Keywords:** crystal structure, di­meth­ylammonium salt, hexa­aqua­cobalt(II) salt, sulfate, hydrogen bonding

## Abstract

The title salt, (C_2_H_8_N)_2_[Co(H_2_O)_6_)](SO_4_)_2_·2H_2_O, is isotypic with (C_2_H_8_N)_2_[Ni(H_2_O)_6_)](SO_4_)_2_·2H_2_O. The Co—O bond lengths in the [Co(H_2_O)_6_]^2+^ complex cation show very similar distances as in the related Tutton salt (NH_4_)_2_[Co(H_2_O)_6_)](SO_4_)_2_ [average 2.093 (17) Å], but are significantly longer than in the isotypic Ni^II^ compound (Δ*d* ≃ 0.04 Å). The cobalt cation reaches an overall bond-valence sum of 1.97 valence units. The S—O distances are nearly equal, ranging from 1.454 (4) to 1.470 (3) Å [mean 1.465 (12) Å]; however, the O—S—O angles vary clearly from 108.1 (2) to 110.2 (2)° [average bond angle 109.5 (9)°]. The non-coordinating water mol­ecules and di­methyl­ammonium cations connect the sulfate tetrahedra and the [Co(H_2_O)_6_]^2+^ octa­hedron *via* O—H⋯O and N—H⋯O hydrogen bonds of weak up to medium strength into a three-dimensional framework whereby the complex metal cations and sulfate anions are arranged in sheets parallel to (001).

## Related literature   

For the synthesis and coordination geometry of the isotypic structure (C_2_H_8_N)_2_[Ni(H_2_O)_6_)](SO_4_)_2_·2H_2_O, see: Held (2014 ▸). For the related Tutton salt (NH_4_)_2_[Co(H_2_O)_6_)](SO_4_)_2_, see: Grimes *et al.* (1963 ▸). For the bond-valence-sum method, see: Brown & Altermatt (1985 ▸).
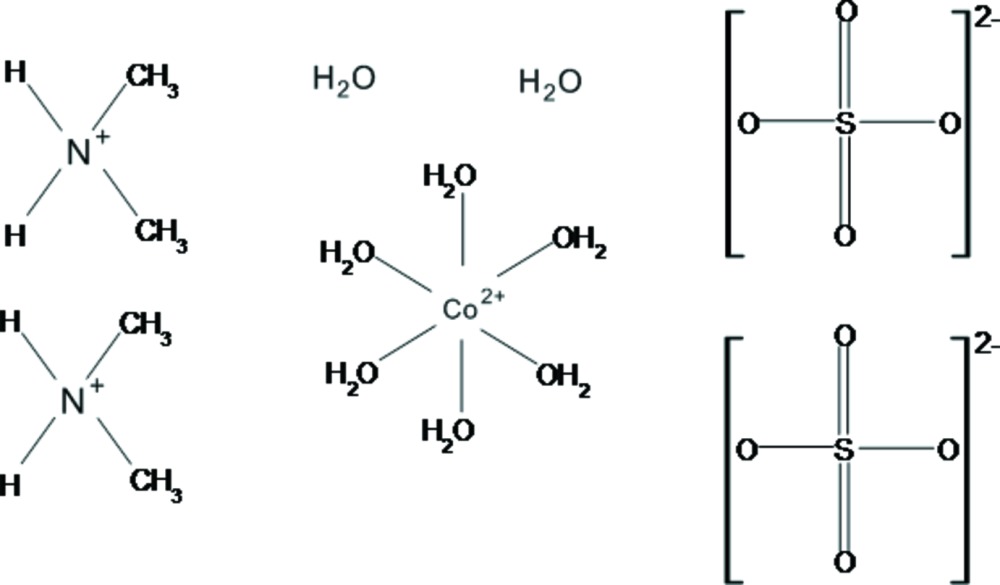


## Experimental   

### Crystal data  

(C_2_H_8_N)_2_[Co(H_2_O)_6_](SO_4_)_2_·2H_2_O
*M* *_r_* = 487.37
Orthorhombic, 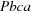

*a* = 8.975 (5) Å
*b* = 13.268 (5) Å
*c* = 16.528 (5) Å
*V* = 1968.2 (15) Å^3^
*Z* = 4
Mo *K*α radiation
μ = 1.16 mm^−1^
*T* = 295 K
0.30 × 0.27 × 0.24 mm

### Data collection  

Enraf–Nonius CAD-4 diffractometer
Absorption correction: ψ scan (North *et al.*, 1968 ▸) *T*_min_ = 0.903, *T*_max_ = 1.000
3383 measured reflections
1733 independent reflections
936 reflections with *I* > 2σ(*I*)
*R*_int_ = 0.077
3 standard reflections every 100 reflections intensity decay: 1.5%

### Refinement  

*R*[*F* ^2^ > 2σ(*F* ^2^)] = 0.039
*wR*(*F* ^2^) = 0.111
*S* = 0.98
1733 reflections
148 parameters
2 restraints
H atoms treated by a mixture of independent and constrained refinement
Δρ_max_ = 0.41 e Å^−3^
Δρ_min_ = −0.37 e Å^−3^

### 

Data collection: *CAD-4 EXPRESS* (Enraf–Nonius, 1989 ▸); cell refinement: *CAD-4 EXPRESS*; data reduction: *MolEN* (Fair, 1990 ▸); program(s) used to solve structure: *SIR97* (Altomare *et al.*, 1999 ▸); program(s) used to refine structure: *SHELXL97* (Sheldrick, 2008 ▸); molecular graphics: *ATOMS* (Dowty, 2011 ▸); software used to prepare material for publication: *SHELXL97* and *publCIF* (Westrip, 2010 ▸).

## Supplementary Material

Crystal structure: contains datablock(s) I, global. DOI: 10.1107/S2056989015003400/fk2085sup1.cif

**Fig. 1.**
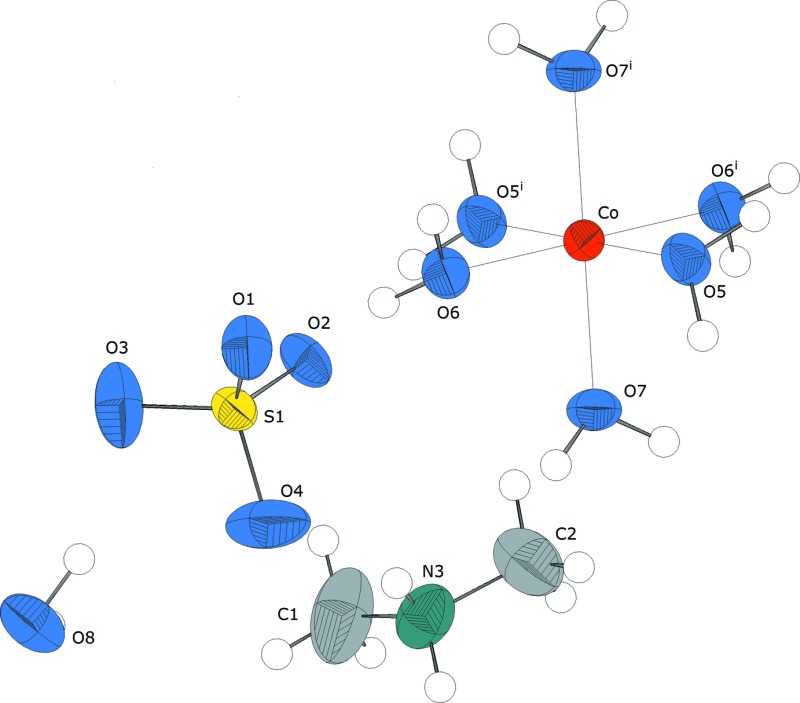
The molecular entities in the structure of the title compound. Displacement ellipsoids are drawn at the 50% probability level. [Symmetry code: (i) -x, -y + 1, -z - 1.]

Structure factors: contains datablock(s) I. DOI: 10.1107/S2056989015003400/fk2085Isup2.hkl

x y z . DOI: 10.1107/S2056989015003400/fk2085fig1.tif
The mol­ecular entities in the structure of the title compound. Displacement ellipsoids are drawn at the 50% probability level. [Symmetry code: (i) −*x*, −*y* + 1, −*z* − 1.]

**Fig. 2.**
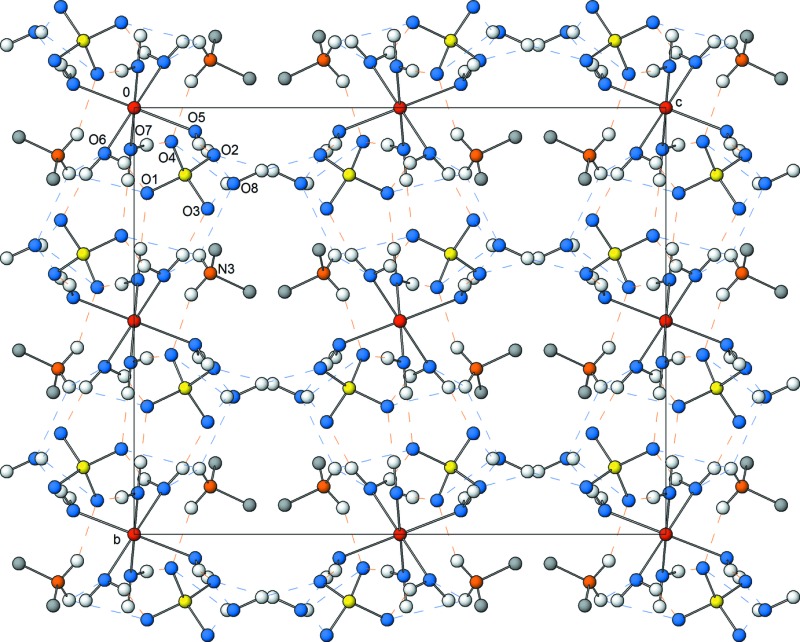
(100)-projection of the crystal structure of the title compound. Colour scheme: S (yellow), Co (red), O (blue), N (orange), C (grey), H (colourless), H···O bonds up to 1.8 Å are given as red dashed lines, and from 1.85 to 2.7 Å as light-blue dashed lines.

. DOI: 10.1107/S2056989015003400/fk2085fig2.tif
(100)-projection of the crystal structure of the title compound. Colour scheme: S (yellow), Co (red), O (blue), N (orange), C (grey), H (colourless), H⋯O bonds up to 1.8 Å are given as red dashed lines, and from 1.85 to 2.7 Å as light-blue dashed lines.

CCDC reference: 1050102

Additional supporting information:  crystallographic information; 3D view; checkCIF report

## Figures and Tables

**Table 1:** Hydrogen-bond geometry (, )

*D*H*A*	*D*H	H*A*	*D* *A*	*D*H*A*
O5H51O2^i^	0.82(6)	1.91(6)	2.724(6)	169(5)
O5H52O8	0.84(7)	1.97(8)	2.806(7)	171(7)
O6H61O3^ii^	0.85(7)	1.85(7)	2.687(6)	173(6)
O6H62O1	0.69(5)	2.08(5)	2.740(6)	161(6)
O7H71O4^iii^	0.74(6)	2.01(6)	2.740(6)	173(6)
O7H72O1^iv^	0.72(4)	2.04(4)	2.756(6)	176(5)
O8H81O3^iii^	0.71(6)	2.32(6)	2.975(7)	154(7)
O8H82O2^v^	0.83(6)	2.02(6)	2.849(6)	169(7)
N3H3*A*O6^iv^	0.90	2.63	3.265(6)	128
N3H3*B*O4^vi^	0.90	2.00	2.823(6)	152

Symmetry codes: (i) 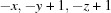; (ii) 
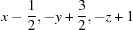
; (iii) 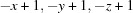; (iv) 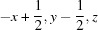; (v) 
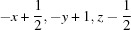
; (vi) 
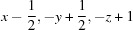
.

## supplementary crystallographic information

### S1. Refinement

All H atoms were clearly discernible from difference Fourier maps. However, to all hydrogen atoms riding model contraints were applied in the least squares refinement, with C—H = 0.96 Å and *U*_iso_(H) = 1.5*U*_eq_(C) for methyl H atoms and with N—H = 0.90 Å and *U*_iso_(H) = 1.2*U*_eq_(N) for ammonium H atoms. The H atoms of water molecules were refined with a distance restraint of O—H = 0.84 Å and individual *U*_iso_ values for each H atom.

### Crystal data

**Table:**

(C_2_H_8_N)_2_[Co(H_2_O)_6_](SO_4_)_2_·2H_2_O	*F*(000) = 1028
*M**_r_* = 487.37	*D*_x_ = 1.645 Mg m^−^^3^
Orthorhombic, *P**b**c**a*	Mo *K*α radiation, λ = 0.71073 Å
Hall symbol: -P 2ac 2ab	Cell parameters from 25 reflections
*a* = 8.975 (5) Å	θ = 12.0–20.8°
*b* = 13.268 (5) Å	µ = 1.16 mm^−^^1^
*c* = 16.528 (5) Å	*T* = 295 K
*V* = 1968.2 (15) Å^3^	Parallelepiped, light blue
*Z* = 4	0.30 × 0.27 × 0.24 mm

### Data collection

**Table:**

Enraf–Nonius CAD-4 diffractometer	936 reflections with *I* > 2σ(*I*)
Radiation source: fine-focus sealed tube	*R*_int_ = 0.077
Graphite monochromator	θ_max_ = 25.0°, θ_min_ = 2.5°
ω/2θ scans	*h* = 0→10
Absorption correction: ψ scan (North *et al.*, 1968)	*k* = 0→15
*T*_min_ = 0.903, *T*_max_ = 1.000	*l* = −19→19
3383 measured reflections	3 standard reflections every 100 reflections
1733 independent reflections	intensity decay: 1.5%

### Refinement

**Table:**

Refinement on *F*^2^	Secondary atom site location: difference Fourier map
Least-squares matrix: full	Hydrogen site location: difference Fourier map
*R*[*F*^2^ > 2σ(*F*^2^)] = 0.039	H atoms treated by a mixture of independent and constrained refinement
*wR*(*F*^2^) = 0.111	*w* = 1/[σ^2^(*F*_o_^2^) + (0.0529*P*)^2^] where *P* = (*F*_o_^2^ + 2*F*_c_^2^)/3
*S* = 0.98	(Δ/σ)_max_ < 0.001
1733 reflections	Δρ_max_ = 0.41 e Å^−^^3^
148 parameters	Δρ_min_ = −0.37 e Å^−^^3^
2 restraints	Extinction correction: *SHELXL*, Fc^*^=kFc[1+0.001xFc^2^λ^3^/sin(2θ)]^-1/4^
Primary atom site location: structure-invariant direct methods	Extinction coefficient: 0.0022 (6)

### Special details

**Table:**

Experimental. A suitable single-crystal was carefully selected under a polarizing microscope and mounted in a glass capillary.
Geometry. All e.s.d.'s (except the e.s.d. in the dihedral angle between two l.s. planes) are estimated using the full covariance matrix. The cell e.s.d.'s are taken into account individually in the estimation of e.s.d.'s in distances, angles and torsion angles; correlations between e.s.d.'s in cell parameters are only used when they are defined by crystal symmetry. An approximate (isotropic) treatment of cell e.s.d.'s is used for estimating e.s.d.'s involving l.s. planes.
Refinement. Refinement of *F*^2^ against ALL reflections. The weighted *R*-factor *wR* and goodness of fit *S* are based on *F*^2^, conventional *R*-factors *R* are based on *F*, with *F* set to zero for negative *F*^2^. The threshold expression of *F*^2^ > σ(*F*^2^) is used only for calculating *R*-factors(gt) *etc*. and is not relevant to the choice of reflections for refinement. *R*-factors based on *F*^2^ are statistically about twice as large as those based on *F*, and *R*- factors based on ALL data will be even larger.

### Fractional atomic coordinates and isotropic or equivalent isotropic displacement parameters (Å^2^)

**Table:**

	*x*	*y*	*z*	*U*_iso_*/*U*_eq_	
Co	0.0000	0.5000	0.5000	0.0278 (3)	
S1	0.44510 (13)	0.65726 (9)	0.59555 (8)	0.0319 (3)	
O1	0.3702 (4)	0.6999 (2)	0.5243 (2)	0.0466 (10)	
O2	0.3355 (4)	0.6107 (3)	0.6497 (2)	0.0419 (9)	
O3	0.5255 (5)	0.7366 (3)	0.6378 (2)	0.0634 (12)	
O4	0.5493 (5)	0.5793 (3)	0.5696 (3)	0.0791 (15)	
O5	−0.0505 (5)	0.4446 (3)	0.3854 (2)	0.0422 (10)	
H51	−0.136 (7)	0.422 (4)	0.380 (3)	0.040 (18)*	
H52	0.010 (8)	0.403 (5)	0.365 (5)	0.11 (3)*	
O6	0.1413 (5)	0.6068 (3)	0.4439 (3)	0.0391 (10)	
H61	0.103 (8)	0.653 (5)	0.415 (4)	0.09 (3)*	
H62	0.198 (6)	0.620 (4)	0.470 (3)	0.03 (2)*	
O7	0.1808 (5)	0.4036 (3)	0.5066 (3)	0.0444 (10)	
H71	0.251 (7)	0.412 (4)	0.484 (3)	0.037 (19)*	
H72	0.172 (5)	0.350 (3)	0.512 (3)	0.021 (16)*	
O8	0.1675 (5)	0.3224 (4)	0.3135 (3)	0.0558 (13)	
H81	0.245 (7)	0.326 (5)	0.322 (4)	0.06 (2)*	
H82	0.174 (8)	0.348 (5)	0.268 (4)	0.09 (3)*	
N3	0.0327 (6)	0.1120 (3)	0.3563 (3)	0.0567 (14)	
H3A	0.1016	0.1541	0.3769	0.068*	
H3B	0.0187	0.0622	0.3925	0.068*	
C1	0.0909 (10)	0.0689 (5)	0.2833 (4)	0.094 (3)	
H1A	0.1825	0.0344	0.2950	0.141*	
H1B	0.1092	0.1214	0.2446	0.141*	
H1C	0.0201	0.0219	0.2615	0.141*	
C2	−0.1060 (7)	0.1669 (5)	0.3473 (4)	0.074 (2)	
H2A	−0.1450	0.1833	0.3998	0.111*	
H2B	−0.1767	0.1260	0.3187	0.111*	
H2C	−0.0883	0.2279	0.3175	0.111*	

### Atomic displacement parameters (Å^2^)

**Table:**

	*U*^11^	*U*^22^	*U*^33^	*U*^12^	*U*^13^	*U*^23^
Co	0.0269 (5)	0.0257 (4)	0.0309 (4)	0.0003 (5)	−0.0026 (5)	−0.0010 (5)
S1	0.0249 (6)	0.0340 (6)	0.0368 (7)	−0.0010 (6)	−0.0005 (6)	0.0065 (6)
O1	0.055 (2)	0.042 (2)	0.044 (2)	−0.0080 (19)	−0.0180 (18)	0.0107 (17)
O2	0.0289 (19)	0.052 (2)	0.045 (2)	−0.0104 (17)	0.0059 (17)	0.0093 (18)
O3	0.084 (3)	0.060 (2)	0.047 (2)	−0.034 (3)	−0.023 (2)	0.0151 (19)
O4	0.063 (3)	0.067 (3)	0.108 (4)	0.026 (2)	0.042 (3)	0.032 (3)
O5	0.034 (2)	0.053 (2)	0.040 (2)	−0.004 (2)	−0.002 (2)	−0.0102 (19)
O6	0.035 (2)	0.039 (2)	0.043 (2)	−0.004 (2)	−0.007 (2)	0.007 (2)
O7	0.035 (2)	0.036 (3)	0.062 (3)	0.007 (2)	0.012 (2)	0.010 (2)
O8	0.035 (3)	0.093 (4)	0.039 (3)	−0.004 (3)	−0.001 (2)	−0.009 (3)
N3	0.069 (4)	0.047 (3)	0.054 (3)	0.005 (3)	−0.018 (3)	−0.004 (2)
C1	0.135 (8)	0.056 (4)	0.090 (6)	0.016 (5)	0.054 (5)	0.009 (4)
C2	0.057 (4)	0.084 (5)	0.080 (5)	0.005 (4)	0.007 (4)	0.016 (4)

### Geometric parameters (Å, º)

**Table:**

Co—O7^i^	2.069 (4)	O7—H71	0.74 (6)
Co—O7	2.069 (4)	O7—H72	0.72 (4)
Co—O5^i^	2.081 (4)	O8—H81	0.71 (6)
Co—O5	2.081 (4)	O8—H82	0.83 (6)
Co—O6	2.116 (4)	N3—C1	1.434 (7)
Co—O6^i^	2.116 (4)	N3—C2	1.450 (7)
S1—O3	1.454 (4)	N3—H3A	0.9000
S1—O4	1.459 (4)	N3—H3B	0.9000
S1—O2	1.467 (3)	C1—H1A	0.9600
S1—O1	1.470 (3)	C1—H1B	0.9600
O5—H51	0.82 (6)	C1—H1C	0.9600
O5—H52	0.84 (7)	C2—H2A	0.9600
O6—H61	0.85 (7)	C2—H2B	0.9600
O6—H62	0.69 (5)	C2—H2C	0.9600
			
O7^i^—Co—O7	180.0 (3)	Co—O6—H62	109 (4)
O7^i^—Co—O5^i^	90.04 (18)	H61—O6—H62	119 (6)
O7—Co—O5^i^	89.96 (18)	Co—O7—H71	123 (4)
O7^i^—Co—O5	89.96 (18)	Co—O7—H72	122 (4)
O7—Co—O5	90.04 (18)	H71—O7—H72	109 (6)
O5^i^—Co—O5	180.000 (1)	H81—O8—H82	95 (7)
O7^i^—Co—O6	91.87 (19)	C1—N3—C2	115.3 (5)
O7—Co—O6	88.13 (19)	C1—N3—H3A	108.5
O5^i^—Co—O6	91.80 (18)	C2—N3—H3A	108.5
O5—Co—O6	88.20 (18)	C1—N3—H3B	108.5
O7^i^—Co—O6^i^	88.13 (19)	C2—N3—H3B	108.5
O7—Co—O6^i^	91.87 (19)	H3A—N3—H3B	107.5
O5^i^—Co—O6^i^	88.20 (18)	N3—C1—H1A	109.5
O5—Co—O6^i^	91.80 (18)	N3—C1—H1B	109.5
O6—Co—O6^i^	180.0	H1A—C1—H1B	109.5
O3—S1—O4	109.6 (3)	N3—C1—H1C	109.5
O3—S1—O2	110.1 (2)	H1A—C1—H1C	109.5
O4—S1—O2	108.1 (2)	H1B—C1—H1C	109.5
O3—S1—O1	109.5 (2)	N3—C2—H2A	109.5
O4—S1—O1	109.3 (3)	N3—C2—H2B	109.5
O2—S1—O1	110.2 (2)	H2A—C2—H2B	109.5
Co—O5—H51	116 (4)	N3—C2—H2C	109.5
Co—O5—H52	117 (5)	H2A—C2—H2C	109.5
H51—O5—H52	108 (6)	H2B—C2—H2C	109.5
Co—O6—H61	119 (5)		

Symmetry code: (i) −*x*, −*y*+1, −*z*+1.

### Hydrogen-bond geometry (Å, º)

**Table:**

*D*—H···*A*	*D*—H	H···*A*	*D*···*A*	*D*—H···*A*
O5—H51···O2^i^	0.82 (6)	1.91 (6)	2.724 (6)	169 (5)
O5—H52···O8	0.84 (7)	1.97 (8)	2.806 (7)	171 (7)
O6—H61···O3^ii^	0.85 (7)	1.85 (7)	2.687 (6)	173 (6)
O6—H62···O1	0.69 (5)	2.08 (5)	2.740 (6)	161 (6)
O7—H71···O4^iii^	0.74 (6)	2.01 (6)	2.740 (6)	173 (6)
O7—H72···O1^iv^	0.72 (4)	2.04 (4)	2.756 (6)	176 (5)
O8—H81···O3^iii^	0.71 (6)	2.32 (6)	2.975 (7)	154 (7)
O8—H82···O2^v^	0.83 (6)	2.02 (6)	2.849 (6)	169 (7)
N3—H3*A*···O6^iv^	0.90	2.63	3.265 (6)	128
N3—H3*B*···O4^vi^	0.90	2.00	2.823 (6)	152

Symmetry codes: (i) −*x*, −*y*+1, −*z*+1; (ii) *x*−1/2, −*y*+3/2, −*z*+1; (iii) −*x*+1, −*y*+1, −*z*+1; (iv) −*x*+1/2, *y*−1/2, *z*; (v) −*x*+1/2, −*y*+1, *z*−1/2; (vi) *x*−1/2, −*y*+1/2, −*z*+1.
