# Supplementary figures and images for: Crystal structure of bis­(di­methyl­ammonium) hexa­aqua­cobalt(II) bis­(sulfate) dihydrate
Source: Acta Crystallogr E Crystallogr Commun. 2015 Mar 4;71(Pt 4):m77–8. doi: 10.1107/S2056989015003400 (PMC4438788; doi:10.1107/S2056989015003400)

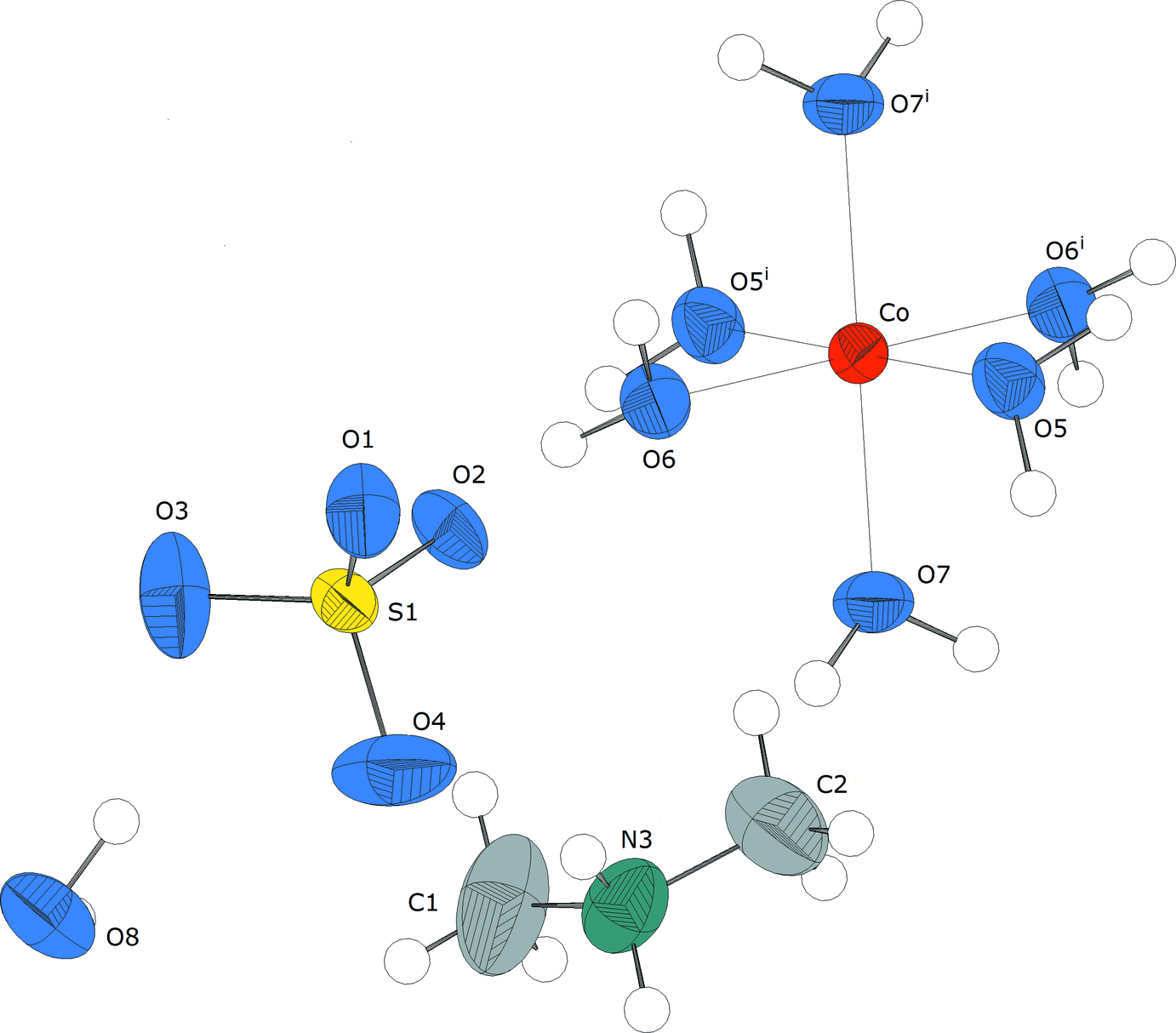

Supplement: Supplementary file 3 [file e-71-00m77-fig1.tif]

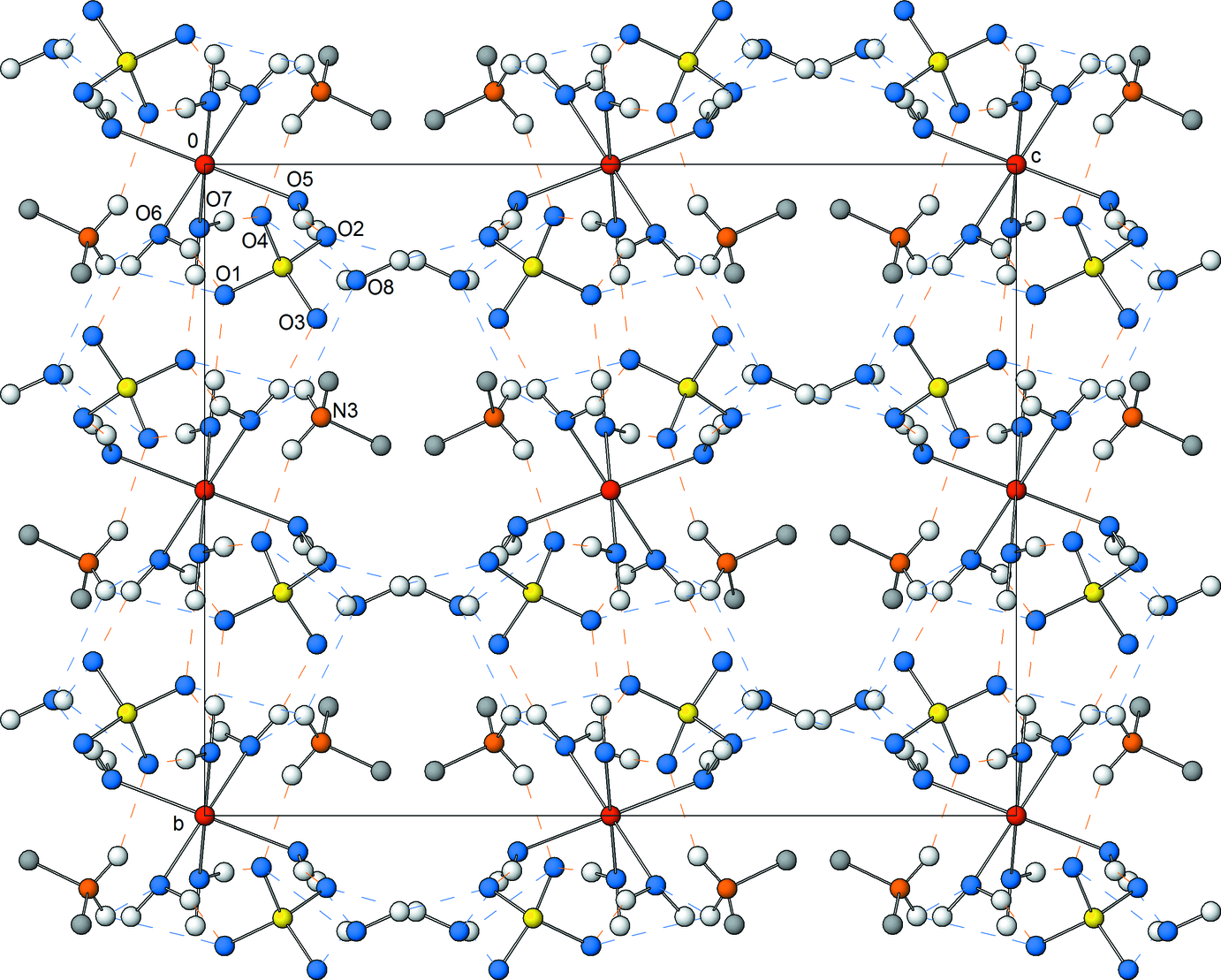

Supplement: Supplementary file 4 [file e-71-00m77-fig2.tif]
